# Hemolytic anemia caused by non-D minor blood incompatibilities in a newborn

**DOI:** 10.11604/pamj.2019.33.262.19324

**Published:** 2019-07-29

**Authors:** Ali Ulas Tugcu, Deniz Anuk Ince, Ozden Turan, Burcu Belen, Lale Olcay, Ayse Ecevit

**Affiliations:** 1 Baskent University Ankara Hospital, Department of Pediatrics, Division of Neonatology, Ankara, Turkey; 2 Baskent University Ankara Hospital, Department of Pediatrics, Division of Pediatric Hematology, Ankara, Turkey

**Keywords:** Hemolytic anemia, minor blood incompability, hyperbilirubinemia

## Abstract

Hyperbilirubinemia is one of the most widely seen cause of neonatal morbidity. Besides ABO and Rh isoimmunization, minor blood incompatibilities have been also been identified as the other causes of severe newborn jaundice. We report a newborn with indirect hyperbilirubinemia caused by minor blood group incompatibilities (P1, M, N, s and Duffy) whose hemolysis was successfully managed with intravenous immunoglobulin therapy. A thirty-two gestational weeks of preterm male baby became severely icteric on postnatal day 11, with a total bilirubin level of 14.66 mg/dl. Antibody screening tests revealed incompatibility on different minor groups (P1, M, N, s and Duffy (Fya ve Fyb)). On postnatal day thirteen, the level of bilirubin increased to 20.66 mg/dl although baby was under intensive phototherapy. After the administration of intravenous immunoglobulin and red blood cell transfusion, hemoglobin and total bilirubin levels became stabilised. Minor blood incompatibilities should be kept in mind during differential diagnosis of hemolytic anemia of the newborn. They share the same treatment algorithm with the other types hemolytic anemia. New studies revealed that intravenous immunoglobulin treatment in hemolytic anemia have some attractive and glamorous results. It should be seriously taken into consideration for treatment of minor blood incompatibilities.

## Introduction

Hyperbilirubinemia is one of the most widely seen cause of neonatal morbidity. Hemolytic disease of newborn (HDN) is an important cause of hyperbilirubinemia. It is defined as incompatibility between maternal and infant blood groups, which results in destruction of fetal red blood cells leading to high bilirubin levels [1]. ABO and Rh incompatibility are the most common causes of severe indirect hyperbilirubinemia. Besides ABO and Rh isoimmunization, minor blood incompatibilities (MBI) such as anti-Kell, anti-C, anti-E, anti- MNS, Duffy, Kidd, P, Lutherian and Lewis, have been also been identified as the other causes of severe newborn jaundice [2]. Hemolysis due to MBI presents with clinical and laboratory results; ranging between mild anemia, reticulocytosis and neonatal hyperbilirubinemia and marked fetal anemia and hydropic changes [3]. Intravenous immunoglobulin (IVIG) has been used as an alternative treatment modality for HDN, as it has been shown to decrease the need for red blood cell transfusion [4]. Here we report a newborn with indirect hyperbilirubinemia caused by minor blood group incompatibility (P1, M, N, s and Duffy) whose hemolysis was successfully managed with IVIG therapy.

## Patient and observation

A 32 gestational week preterm male baby, weighing 1815 gr was born with c/s, to 32 year old lady and was transferred to the neonatal intensive care unit. The mother's blood group was O Rh (+) and the baby's blood group was found out to be O Rh(-). Prenatal history was unremarkable. On postnatal (PN) day 4, total bilirubin was detected as 10.8 mg/dl. The level increased to 14.6 mg/dl on PN day 5 (direct bilirubin: 0.76 mg/dl). Complete Blood Count (CBC) revealed a hemoglobin (Hb) of 15.4 gr/dl and hematocrit of 46, as % of reticulocyte is 1.6. Liver function tests (LFT) were normal and direct coombs test was negative. There were no signs of hemolysis at the peripheral smear. Phototherapy was initiated according to American Academy of Pediatric (AAP) normogram. The baby became icteric again on PN day 11, with a total bilirubin level of 14.66 mg/dl and reticulocyte level of 8.7%. LFTs were normal. Target cells and fragmented erythrocytes were seen at the peripheral smear. Sepsis work-up was found to be negative. Antibody screening and defining tests revealed incompatibility on different minor groups (P1, M, N, s and Duffy (Fya ve Fyb)) On postnatal day 13, the level of bilirubin reached to 20,66 mg/dl although baby was under continuous and intensive phototherapy. There wasn't any clinical sign of bilirubin encephalopathy. Repeated CBC examinations revealed a decrease in Hb (8.6 gr/dl) and reticulocyte count of 7.8%. The administration of 1 gr/kg of IVIG and 15 ml/kg red blood cell transfusion was established. Hb levels gradually increased to 11.2 gr/dl and the level of total bilirubin levels became stabilised between 8 -10 mg/dl after IVIG therapy, transfusion and three day long-phototherapy. The baby was discharged with full recovery on PN day 27.

## Discussion

Hemolytic disease of newborn is the most frequent cause of pathologic jaundice. ABO and Rh incompatibilities are the most known and frequent causes of hemolysis. Till date, 49 Rh antigens have been determined. D, C, E, c, e, Duffy, Kidd, MNS are among the most significant ones that were determined. Disease spectrum for non-anti-D erythrocyte alloimmunization depends on the type of antigen and degree of hemolysis. It has been reported that the most severe hemolytic clinic is pictured by Anti c antibodies [5]. Several strategies have been developed to prevent D immunization, leading to a substantial decrease of D immunization in many countries [6]. Consequently, alloantibodies other than anti-D emerged as an important cause of severe HDN after prevention of D immunization. Many minor blood group systems were identified since 1927. The diagnosis and treatment process of minor blood incompatibilities are usually delayed. During the diagnosis phase of HDN, most of the clinicians do not give priority to minor blood groups, because hemolysis caused by MNI do not have any specific treatment modality than other incompatibilities [6]. P minor blood group also known as was first identified by Landsteiner in 1927. This system is nowadays renamed and assumed as the part of P1PK blood system [7]. MNS blood antigens are defined in 1927. 30% of all population is negative for antigen M and are capable of producing Anti M when exposed to antigen. However, the incidence of severe HDN due to anti M antibodies is rarely reported. De Young-Owens A *et al.* showed no cases of hemolytic disease of the newborn, mild or severe; in a data collected from a total of 115 pregnancies [8].

Antigen Fya is classified in Duffy system. It may enhance the formation of severe hemolysis in newborns, because it has strong capacity to set off antibodies. In contrary to this, antigen Fyb, which is also classified in Duffy system, is rarely reported to cause hemolysis [9]. Anti S and Anti s are speculated to cause hemolysis during postnatal period, but there is no enough report supporting this claim. We evaluated the hemolytic process of our patient on PN day 11. After check for the differential diagnosis, incompatibilities in minor blood groups were determined (P1, M, N, s, Duffy (Fya and Fyb). We argued that, all the incompatibilities defined in minor blood group research, might promote the hemolytic anemia in our patient. However, most of the reports in literature about HA due to MBI, usually refer to single antibody-antigen incompatibility. Minor blood group incompatibilities share the same treatment modality with other blood group incompatibilities. Jaundice caused by hemolysis is treated with phototherapy. Hemolytic anemia with hydrops may need transfusion with sub-group matched RBCs. Some few reports are available about the usage of IVIG in hemolytic anemia caused by minor blood incompatibilities [9]. IVIG treatment decreases the need for RBC transfusion for hemolytic anemia, but the exact mechanism is not clearly identified yet [10]. We administrated 1 gr/kg IVIG and RBC transfusion. After IVIG treatment, Hb levels gradually started to increase, in accordance with a progressive reduction in bilirubin levels. No exchange transfusion was required.

## Conclusion

MBI should be kept in mind during differential diagnosis of hemolytic anemia of the newborn. They have the same treatment algorithm with the other types of hemolytic anemia. This includes phototherapy, IVIG and transfusion. New studies revealed that IVIG treatment in HA have some attractive and glamorous results. It should be seriously taken into consideration for treatment of MBI.

## Competing interests

All authors declare no competing interests.
